# Approach–Avoidance Motivation and Goal Adaptation in Chronic Pain: Predicting Pain Intensity and Interference

**DOI:** 10.3390/ijerph23060708

**Published:** 2026-05-27

**Authors:** Paria Eshraghi, Deirdre Drake, Joanne Iddon, Joanne M. Dickson

**Affiliations:** 1 School of Arts & Humanities, Psychology Discipline, Edith Cowan University, Joondalup 6027, Australia; zeshragh@our.ecu.edu.au (P.E.); d.drake@ecu.edu.au (D.D.); 2 Mental Health Research Group, Centre for Precision Health, Edith Cowan University, Joondalup 6027, Australia; 3 Mersey Care NHS Foundation Trust, Kings Business Park, Trust Offices, V7 Building, Prescot L34 1PJ, UK; joanne.iddon@merseycare.nhs.uk

**Keywords:** chronic pain, avoidance motivation, approach motivation, goal disengagement, goal re-engagement, goal flexibility, goal tenacity, solving pain, meaningfulness of life, acceptance of the insolubility of pain

## Abstract

**Highlights:**

**Public health relevance—How does this work relate to a public health issue?**

- Chronic pain is a major public health burden associated with disability and psychological distress.
- This study highlights chronic pain as disrupting personal goals and daily roles, demonstrating the importance of motivational and goal-related processes implicated in functioning and well-being.

**Public health significance—Why is this work of significance to public health?**

- Chronic pain is often not fully resolvable for individuals. Motivation and adaptive goal processes extend our understanding of how some individuals maintain optimal functioning despite pain.
- Avoidance motivation predicted higher pain intensity and interference, whereas approach motivation did not. Goal disengagement and meaningfulness of life predicted lower pain outcomes, despite pain.

**Public health implications—What are the key implications or messages for practitioners, policymakers and/or researchers in public health?**

- The present research highlights the importance of clinical assessments for chronic pain that include psychological factors such as motivation and adaptive goal processes and strategies, alongside routine practice.
- The findings suggest that person-centred chronic pain approaches should consider motivational and goal regulation processes relevant to adjustment and daily functioning.

**Abstract:**

Chronic pain is a major public health concern associated with disability, reduced functioning and psychological distress. Compared with biological studies, relatively little research has studied chronic pain from a psychological perspective, specifically motivation. Yet, motivation is considered fundamental to human functioning and drives human behaviour. The present study aimed to investigate whether distinct motivational and adaptive goal processes are implicated in chronic pain. The sample comprised 190 adult community participants who reported chronic pain for 3 months or more. Participants completed self-report measures of approach and avoidance motivation, adaptive goal processes, pain intensity, and interference. As hypothesised, avoidance motivation (but not approach motivation) was positively and significantly associated with increased pain intensity and interference. Further, increased goal disengagement was negatively and significantly related to reduced pain intensity and interference, as predicted. Contrary to expectation, goal re-engagement was positively related to increased pain interference. As predicted, solving pain was significantly and positively associated with pain intensity and interference. As predicted, meaningfulness of life was related to lower pain interference but not pain intensity. Counter to prediction, acceptance of the insolubility of pain was significantly and positively related to both pain intensity and pain interference. Our findings highlight significant associations between motivational orientation, adaptive goal regulation processes, and chronic pain intensity and interference and underscore the importance of understanding chronic pain from a motivational perspective.

## 1. Introduction

Chronic pain, typically defined as pain that persists or recurs beyond normal healing time and lasts for longer than three months [1], is a widespread and debilitating condition that affects approximately one in five adults globally [1,2,3] and represents a major public health challenge. Chronic pain is a leading cause of long-term disability [2,4] and significantly impairs individuals’ ability to engage in work, social events, and everyday activities [5,6]. In addition to its individual impact, chronic pain imposes substantial economic costs through reduced productivity, absenteeism, and increased healthcare expenditure [7]. In Australia, particularly, the total financial costs of chronic pain were reported as AUD 144 billion in 2020, reflecting not only healthcare and productivity losses but also the broader impact on quality of life [8]. Chronic pain is widely recognised as a complex biopsychosocial phenomenon, shaped not just by biological factors but also by psychological and social influences [9,10]. Psychological consequences are common, with individuals often experiencing depression, anxiety [11,12], and reduced mental well-being [13]. The “invisible” nature of pain contributes to social stigma and discrimination [14,15], which can undermine identity and increase social withdrawal [16]. Chronic pain has also been linked to an increased risk of suicidal ideation and mental defeat [17], as well as substance use disorders [6,18].

Despite advances in pain research and treatment, many individuals continue to live with chronic pain in their daily lives. Contemporary clinical approaches have increasingly shifted from aiming to eliminate pain toward promoting optimal functioning and psychosocial adjustment [4]. Despite medical advances, complete pain relief is often unattainable, with growing emphasis instead placed on effective management to support individuals living well alongside their pain [19]. This shift has fuelled increased interest in psychological factors that may influence adaptation to chronic pain. In particular, motivational orientations and goal-related processes have emerged as relatively new and promising areas of research that may help explain why some individuals adapt more effectively than others. The present study aims to understand the nature of pain from the perspective of distinct personal motivation and goal processes.

Gray’s [20] early prominent theoretical model proposes that human behaviour is guided by two fundamental motivational systems, approach and avoidance. The motivational orientations are underpinned by two neurobiological systems: the behavioural activation system (BAS) and the behavioural inhibition system (BIS). High BAS sensitivity represents heightened sensitivity to reward cues and is thought to elicit positive emotions such as happiness, hope, and elation [21,22]. Perceived slow or blocked progress toward a desirable end-state (low BAS) may give rise to frustration, disappointment, and depression [22]. In contrast, the BIS is sensitive to cues of punishment or threat and is associated with heightened vigilance, anxiety, and fear, whereas successful avoidance of aversive cues or outcomes may elicit feelings of relief or calmness [22]. Building on Gray’s framework, self-regulation models further explain how motivational systems guide behaviour toward desired outcomes or away from potential threats. For example, Carver and Scheier’s [22] self-regulation model conceptualises behaviour as monitoring progress toward desired states and adjusting actions accordingly. Similarly, regulatory focus theory distinguishes between promotion-focused motivation oriented toward gains and prevention-focused motivation oriented toward safety and loss avoidance [23]. Approach and avoidance motivational orientations, therefore, reflect general tendencies to move toward desirable outcomes or away from perceived threats [21,24]. Empirical evidence in non-pain samples indicates that stronger avoidance tendencies are associated with greater anxiety and depressive symptoms, whereas stronger approach tendencies are linked to reduced depression and better well-being [25,26,27]. These motivational sensitivities may be particularly relevant for understanding behavioural responses in chronic pain contexts.

To prevent pain exacerbation, individuals with stronger avoidance motivation may withdraw from physical or social activities, such as avoiding movement to reduce the risk of further pain or injury. Although this strategy may provide short-term relief, persistent avoidance is theorised to contribute to the well-established “fear-avoidance cycle”, in which fear of pain leads to activity restriction, physical deconditioning, and ultimately increased disability [4,28,29]. In contrast, it could be argued that individuals with stronger approach motivation may remain engaged in valued activities and continue pursuing meaningful goals despite ongoing pain. For instance, a person experiencing chronic pain may set a goal of completing a 10 min walk several times per week to maintain mood and activity, using pacing strategies and rest breaks to manage discomfort. Such engagement may help buffer against demoralisation and support psychological well-being. Research in chronic pain suggests avoidance-related constructs have been consistently associated with pain-related distress [4,29,30,31]. In contrast, far less research has studied the potential adaptive implications of approach sensitivity in relation to pain. Accordingly, the first aim of this study was to examine the associations between approach and avoidance motivational orientations in relation to pain intensity and pain interference (i.e., the extent to which pain disrupts everyday functioning and roles).

Within motivational systems, personal goals represent an important component within motivation hierarchies [32,33], as personal goals direct and energise behaviour across situations [34,35]. Chronic pain often interferes with the pursuit of valued goals, from everyday tasks to long-term aspirations, by depleting time, energy, and functional capacity [5,36]. In response, individuals may engage in self-regulatory goal adaptation processes to manage these disruptions. Therefore, understanding distinct adaptive goal processes may shed light on maintaining function and psychological well-being when goal pursuit is disrupted or blocked. Adaptive goal processes, including goal disengagement, goal re-engagement, goal flexibility, and goal tenacity, refer to how individuals regulate their goals in response to persistent challenges. The goal adjustment model [37] proposes that adaptive functioning represents the ability to effectively disengage from unattainable goals (goal disengagement) and to re-engage with new meaningful goal alternatives (goal re-engagement).

The dual-process model distinguishes between goal tenacity, defined as sustained effort toward valued goals despite obstacles, and goal flexibility, defined as the ability to adjust or reformulate goals in response to situational constraints [38,39]. Following this dual-process framework, these self-regulatory processes may also be expressed in chronic pain through pain-specific goal strategies [40]. Specifically, assimilative strategies, such as the persistent efforts to solve pain or the belief that a treatment or cure for pain exists, reflect a tenacious focus on pain reduction. In contrast, accommodative strategies, including maintaining meaningfulness of life despite pain and accepting its insolubility, reflect flexible goal adjustment processes that may support adaptive functioning even when pain persists [40]. In chronic pain contexts, where pain is often persistent and not fully resolvable, continued pain control striving may be less adaptive and less likely to reduce pain-related outcomes. By contrast, accommodative strategies may be more conducive to adjustment despite ongoing pain [40,41,42].

Previous research suggests that adaptive goal processes are associated with increased well-being and better psychological outcomes in non-pain samples. For example, goal disengagement and goal re-engagement have been associated with increased subjective well-being [37,43] and greater quality of life [44]. Goal flexibility and goal tenacity have been associated with reduced depressive symptoms [38,45] and better mental well-being [46]. In chronic pain populations, goal disengagement and re-engagement have been associated with better psychological well-being and optimism [47,48]. Goal flexibility and tenacity have likewise been associated with better mental well-being and reduced distress [15,49]. Following this framework, and in relation to pain-specific goal strategies, research suggests that assimilative coping is associated with greater affective distress [40], disability, attentional focus on pain, and catastrophising [41]. In contrast, accommodative coping is linked to lower distress and better functional adjustment [40,42]. However, little research has studied these adaptive goal constructs, alongside pain-specific goal strategies, that may be particularly relevant in chronic pain, where persistent pain often disrupts goal pursuit and daily roles and activities.

Accordingly, the second aim of this study focused on examining whether specific adaptive goal processes (i.e., goal flexibility, tenacity, disengagement, and re-engagement) and pain-specific goal strategies (solving pain, belief in a solution, meaningfulness of life despite pain, and acceptance of the insolubility of pain) are implicated in pain intensity and pain interference.

### Research Hypotheses

Based on the theoretical models of motivation and self-regulation, and past empirical research, we hypothesised that increased avoidance motivation (but not approach motivation) would independently and significantly predict higher pain intensity and pain interference. Next, we hypothesised that adaptive goal processes (i.e., goal flexibility, goal tenacity, goal disengagement, and goal re-engagement) would independently and significantly predict lower reported pain intensity and pain interference. Finally, we hypothesised that accommodative pain-specific goal strategies (i.e., meaningfulness of life despite pain and acceptance of the insolubility of pain), but not assimilative pain-specific goal strategies (i.e., solving pain and belief in a solution), would independently and significantly predict lower pain intensity and pain interference.

## 2. Materials and Methods

### 2.1. Participants and Sample Size

In this study, 190 adult participants (96 women, 94 men) aged 18–88 years (M = 49.46, SD = 17.38), residing in Australia, and experiencing chronic pain volunteered to participate. Chronic pain is defined as pain persisting for three months or more [50]. Consistent with the ICD-11/IASP chronic pain framework [50], broad chronic pain inclusion criteria were adopted, particularly given the relatively novel nature of examining chronic pain from a motivational and goal regulation perspective.

A priori power analysis based on Cohen’s [51] recommendation indicated that 84 participants were required to detect a medium effect size (r = 0.30) with 80% power at *p* < 0.05. The final sample (N = 190) exceeded this requirement and also surpassed recommended guidelines for multiple regression analyses (50 + 8 m cases [52]), which indicated a minimum of 130 participants for the model with the largest number of predictors. Table 1 presents the demographic characteristics of the sample.

The demographic data outlined above shows that over half the participants were in paid employment (full- or part-time), indicating a largely working or otherwise active chronic pain sample.

### 2.2. Measures

**Demographic data:** In addition to the demographics reported above in Table 1, pain characteristics including pain duration (i.e., how long participants had experienced their current pain) and pain type (e.g., musculoskeletal, neuropathic, or other as reported by participants) were measured.

For ease of reference, Table 2 below lists the study measures used, followed by detailed descriptions of each measure.

**The Reinforcement Sensitivity Theory of Personality Questionnaire—Short** (RST-PQ-S [53]). The RST-PQ-S measure assesses approach and avoidance motivational traits. The original measure comprised 65 items [57]. The present study used the abbreviated 22-item version. This scale assesses three fundamental motivational systems: behavioural inhibition system (BIS; 5 items, e.g., “I often worry about letting down other people”), fight–flight–freeze system (FFFS; 5 items, e.g., “I would freeze if I was on a turbulent aircraft”), and behavioural approach system (BAS; 12 items). The BAS comprises four subscales: reward interest (3 items, e.g., “I regularly try new activities just to see if I enjoy them”), goal-drive persistence (3 items, e.g., “I am very persistent in achieving my goals”), reward reactivity (3 items, e.g., “Good news makes me feel overjoyed”), and impulsivity (3 items, e.g., “I often do risky things without thinking of the consequences”). Participants rate each item on a 4-point Likert scale (1 = Not at all, 4 = Highly), with subscale scores for the BIS ranging from 5 to 20, the BAS total scores ranging from 12 to 48, and FFFS scores ranging from 5 to 20 [53]. Consistent with past research [58,59], in the present sample, internal consistency coefficients were 0.70 and 0.85 for BIS and BAS total scores, respectively. The BAS subscale coefficients were 0.75 (reward interest), 0.78 (goal drive persistence), 0.65 (reward reactivity), 0.69 (impulsivity), and 0.56 (FFFS).

**The Tenacious Goal Pursuit and Flexible Goal Adjustment Scales** (TEN/FLEX [38]). The TEN/FLEX assesses adaptive responses to goal-related challenges. The measure comprises two 15-item subscales: tenacious goal pursuit (TGP) (e.g., “When faced with obstacles, I usually double my efforts”) and flexible goal adjustment (FGA) (e.g., “I usually have no difficulties in recognizing where my limits are”). Items are rated on a 5-point Likert scale (1 = strongly disagree, 5 = strongly agree), with total scores ranging from 15 to 75 per subscale. TGP includes nine reverse-scored items, while FGA has four reverse items [60]. Consistent with past research [15,61], the present study reported acceptable reliabilities for both subscales (α = 0.81 for tenacious goal pursuit and α = 0.78 for flexible goal adjustment).

**The Goal Adjustment Scale** (GAS [37]). The GAS assesses an individual’s ability to adjust their goals in response to unattainable goals. It comprises two subscales: goal disengagement (4 items) (e.g., “It is easy for me to reduce my effort toward the goal”) and goal re-engagement (6 items) (e.g., “I think about other new goals to pursue”). Items are rated on a 5-point Likert scale (1 = almost never true, 5 = almost always true), with total scores ranging from 4 to 20 for goal disengagement and 6 to 30 for goal re-engagement. Two items in the goal disengagement subscale are reversely scored. Consistent with past research [37,62,63], the present study reported acceptable reliabilities 0.68 for goal disengagement and 0.90 for goal re-engagement.

**The Pain Solutions Questionnaire** (PaSol [40]) is conceptually grounded in Brandtstädter’s dual-process framework, assessing assimilative (persistent efforts to control pain) and accommodative (adjustment and acceptance) goal coping strategies in response to chronic pain. The PaSol is a 14-item measure, comprising four subscales designed to assess solving pain (4 items; e.g., “I keep searching for ways to control my pain”), meaningfulness of life despite pain (5 items; e.g., “Even when I have severe pain, I still find my life meaningful”), acceptance of the insolubility of pain (3 items; e.g., “I can accept that I can’t control my pain”), and belief in a solution (2 items; e.g., “I have confidence that they will find a solution for my pain”). Participants rate each item on a 7-point Likert scale (0 = Not at all applicable, 6 = Highly applicable). Higher scores indicate greater persistence in problem solving (solving pain), a stronger sense of life meaning despite pain (meaningfulness), greater acceptance of pain’s insolubility (acceptance), and stronger belief in an external solution (belief in a solution). Consistent with past research [40,41], the present study reported acceptable reliabilities were 0.81 (solving pain), 0.86 (meaningfulness of life despite pain), 0.81 (acceptance of the insolubility of pain), and 0.81 (belief in a solution).

**The Chronic Pain Grade Scale** (CPGS [55]) is a 7-item self-report measure of chronic pain severity. In the present study, the pain intensity and pain interference subscales were used to assess these respective pain outcomes. Pain intensity was assessed with three items measuring current, worst, and average pain over the past three months, whereas pain interference was assessed with three items measuring interference in daily functioning, social or recreational activities, and work, including housework. A seventh item assessed the number of days in the past three months that pain prevented usual activities. All items were rated on 0–10 numerical scales. Pain intensity and pain interference scores were calculated by averaging the relevant items and multiplying the mean by 10, yielding subscale scores from 0 to 100. Based on the scoring instruction [56], the CPGS also classifies individuals into four hierarchical pain grades (Grade I: low disability–low intensity; Grade II: low disability–high intensity; Grade III: high disability–moderately limiting; Grade IV: high disability–severely limiting), which were used for descriptive purposes in this study. The CPGS has demonstrated good internal consistency and validity across clinical and community samples. The present study showed sound internal consistency coefficients 0.83 for pain intensity and 0.90 for pain interference.

**The Numeric Rating Scale** (NRS [54]). The NRS is a widely used single-item self-report measure to assess current pain intensity in clinical and research settings. Participants rate the intensity of their current pain on an 11-point scale ranging from 0 (“no pain”) to 10 (“pain as bad as you can imagine”). As a single-item measure, internal consistency reliability (Cronbach’s α) is not applicable. However, the NRS has demonstrated strong construct validity and excellent test–retest reliability in clinical pain populations [64,65].

**The Pain Disability Index** (PDI [55]) is a 7-item self-report instrument designed to assess the degree to which chronic pain interferes with an individual’s participation in essential daily life activities. The PDI assesses the impact of pain in seven domains: family/home responsibilities, recreation, social activity, occupation, sexual behaviour, self-care, and life support activities. The total PDI score is used as an index of pain interference. Each domain is rated on a 0–10 scale, where 0 indicates no disability and 10 indicates total disability; items are summed to yield a total score from 0 to 70, with higher scores reflecting greater pain-related disability. Consistent with past research [55,66], this research showed acceptable reliability in the present sample for the PDI (0.87).

### 2.3. Procedure

Ethics approval for this study was obtained from Edith Cowan University’s Human Research Ethics Committee (HREC No: [2024-05080-DICKSON]). An adult community sample from Australia was recruited through an established online panel provider. Participation was entirely voluntary. Before beginning the survey, participants read a detailed information letter and provided informed consent. Participants were informed that they could withdraw at any time before submitting their responses. All data were collected anonymously. The self-report questionnaire was administered online via Qualtrics and required approximately 20 min to complete. Demographic items were presented first, followed by measures of goal processes, motivational orientations, and pain-related variables. The pain-related variables were presented last to minimise potential mood-priming effects [67].

### 2.4. Data Analysis

Analyses were conducted in SPSS (Version 30). Preliminary correlations examined associations among the main study variables. A series of four separate hierarchical multiple regression analyses were conducted to examine whether motivational orientations and goal-related variables predicted pain intensity and pain interference, respectively, while controlling for age (centred) and gender (0 = male, 1 = female). In the motivation model, age and gender were entered as covariates at Step 1. The three main predictors (BIS, BAS, and FFFS) were entered in Step 2. For goal model, age and gender were entered as covariates at Step 1. Adaptive goal processes (tenacious goal pursuit, flexible goal adjustment, goal disengagement, and goal re-engagement) were entered at Step 2, followed by pain-specific goal strategies (solving pain, meaningfulness of life despite pain, acceptance of the insolubility of pain, and belief in a solution) at Step 3 in relation to pain intensity and pain interference, respectively. Age was mean-centred to reduce non-essential multicollinearity and facilitate interpretation of regression coefficients [52,68]. Model fit was evaluated using *R^2^*, adjusted *R^2^*, and Δ*R^2^*, and standardised *β* coefficients were reported.

## 3. Results

### 3.1. Data Screening

Data were screened for missing data and outliers, and assumptions for parametric analyses were tested. No missing data were observed, as all survey items required a response. No univariate outliers were identified, with all z-scores within ±3.29 [52], except one case for flexible goal adjustment (z = −3.42), which did not change the main effects, so it was retained. Regression diagnostics indicated no violations of parametric assumptions related to normality, linearity, homoscedasticity, multicollinearity, or multivariate outliers across models. Visual inspection of histograms, Q-Q plots, and residual plots supported approximate normality, linearity, and homoscedasticity. No significant multivariate outliers were identified (all Cook’s distances < 1). Regarding the motivational orientation models, multicollinearity was accepted (tolerance = 0.731–0.905; VIF = 1.01–1.37). For the goal process and PaSol models, multicollinearity statistics were also within acceptable ranges (tolerance = 0.463–0.916; VIF = 1.09–2.16).

Preliminary independent-samples t-tests showed significant gender differences for the following variables: NRS pain intensity, BIS, BAS reward reactivity, FFFS, and accommodative goal strategy of acceptance of the insolubility of pain (all *p*’s < 0.05). Age was significantly and negatively correlated with BIS, BAS, and belief in a solution (assimilative goal strategy). Accordingly, gender and age were included as covariates in the regression analyses.

### 3.2. Descriptive Pain Characteristics of the Sample

Table 3 summarises the pain characteristics of the sample.

As can be seen in Table 3, most participants reported musculoskeletal pain as their primary pain condition, with many also experiencing headache/migraine and neuropathic or inflammatory pain. Pain duration at the early stage (3–6 months) and long-standing pain (>5 years) were the most common, together accounting for over half of the sample. Most participants (80.4%) fell within the low-to-moderate and moderate-to-severe pain intensity categories. For descriptive purposes, chronic pain grades were calculated according to the standard scoring procedure [56] and reported as lower disability (Grades I–II) or higher disability (Grades III–IV). Mean disability days were 21.57 (SD = 38.90).

### 3.3. Descriptive Statistics of Main Study Variables

Descriptive statistics for all study variables are presented in Table 4.

### 3.4. Associations Between Pain, Motivation and Adaptive Goal Processes

Preliminary correlations examined associations between pain intensity, pain interference and the main motivation and goal variables (see Table 5).

As expected, avoidance motivation (BIS) was positively and significantly associated with all four pain indices (NRS and CPGS pain intensity; PDI and CPGS pain interference), indicating that increased avoidance motivation was associated with greater pain. In contrast, most BAS subscales were not associated with pain variables, although the BAS impulsivity subscale showed some associations with pain measures.

For goal processes, as expected, tenacious goal pursuit was significantly negatively associated with pain interference (PDI), and flexible goal adjustment was significantly negatively associated with NRS pain intensity, although both effects were small. Regarding pain-specific goal strategies, solving pain showed a small significant positive association with CPGS pain intensity, whereas acceptance of the insolubility of pain was significantly positively associated with both pain intensity and pain interference. All other associations between goal variables, pain outcomes, and the other study constructs were non-significant.

### 3.5. Motivational and Goal Predictors in Relation to Pain Intensity and Interference

A series of four hierarchical regressions were used to test whether motivational orientations predicted (i) pain intensity and (ii) pain interference, respectively, and whether personal adaptive goal processes predicted (i) pain intensity and (ii) pain interference, respectively. Indices of pain intensity and pain interference (CPGS) were used as the primary outcomes of the respective dependent outcome variables. Further analyses based on the alternative pain outcomes (NRS pain intensity and PDI pain interference), reported in the Supplementary Material, showed a comparable pattern of findings.

### 3.6. Motivational Orientations in Relation to Pain Intensity

As predicted, higher avoidance (BIS), but not approach motivation (BAS), uniquely and significantly predicted greater pain intensity. Counter to prediction, flight/fight/freeze (FFFS) did not independently significantly associate with increased pain intensity. Age was also a significant independent predictor in Step 2. Full regression coefficients are presented in Table 6.

### 3.7. Motivational Orientations in Relation to Pain Interference

Hierarchical regression indicated that age and gender were not significant in Step 1. When motivational orientations (BIS, BAS, and FFFS) were entered in Step 2, the model significantly improved and explained significant additional variance in pain interference. As in the previous analysis regarding pain intensity, higher avoidance motivation (BIS), but not approach motivation (BAS), uniquely and significantly predicted greater pain interference. There were no other significant associations. Full regression coefficients are presented in Table 7.

### 3.8. Adaptive Goal Processes and Strategies in Relation to Pain Intensity

The next hierarchical regression was used to examine whether distinct adaptive goal processes (tenacious goal pursuit, flexible goal adjustment, goal disengagement, goal re-engagement) and pain-specific goal strategies (solving pain, meaningfulness of life despite pain, acceptance of the insolubility of pain, and belief in a solution) predicted pain intensity.

As can be seen in Table 8, the covariates age and gender were not significant independent predictors of pain intensity at Step 1. Entering goal process variables (tenacity, flexibility, disengagement, re-engagement) in Step 2 significantly improved the model. However, only goal disengagement significantly and negatively predicted pain intensity. That is, greater goal disengagement predicted less pain intensity. Entering the pain-specific goal strategies at Step 3 produced a further significant increase in explained variance. At this step, counter to prediction, higher acceptance of the insolubility of pain was significantly associated with higher pain intensity. As predicted, a higher focus on eliminating pain, ‘solving pain’, was also significantly associated with higher pain intensity. As predicted, in Step 3, greater goal disengagement remained a significant predictor of lower pain intensity. Tenacious goal pursuit, although non-significant, was approaching a significant negative association with pain intensity. All remaining associations were non-significant.

### 3.9. Adaptive Goal Processes and Strategies in Relation to Pain Interference

As can be seen in Table 9, age and gender were not significant independent predictors of pain interference at Step 1. Entering adaptive goal process variables (tenacity, flexibility, disengagement, re-engagement) in Step 2 significantly improved the model. In Step 3, entering the goal strategies variables significantly improved the model. As can be seen in Step 3, and counter to prediction, greater goal re-engagement and higher acceptance of the insolubility of pain were significantly associated with higher pain interference. As predicted, a greater solving pain (focus on eliminating pain) was significantly associated with higher pain interference, whereas greater meaningfulness of life, despite pain, and greater goal disengagement were significantly associated with lower pain interference. All remaining associations were non-significant.

## 4. Discussion

The present study examined associations and predictors of pain intensity and pain interference across approach and avoidance motivational orientations, adaptive goal processes and strategies in individuals living with chronic pain. Consistent with the hypotheses, we found higher avoidance motivation (BIS), but not approach motivation (BAS), predicted higher pain intensity and pain interference. As predicted, higher goal disengagement predicted lower pain intensity and interference. Contrary to prediction, however, higher goal re-engagement predicted increased pain interference but not pain intensity. As predicted, accommodative strategies of higher meaningfulness of life despite pain predicted lower pain interference but not pain intensity. Contrary to prediction, higher acceptance of the insolubility of pain (accommodative strategy) predicted higher pain intensity and pain interference. As predicted, solving pain (focusing on zero pain) predicted higher pain intensity and pain interference. No other adaptive goal processes were significant predictors of pain intensity or interference.

The avoidance motivation and pain outcomes findings are consistent with chronic pain models that emphasise threat sensitivity [4,29]. Avoidance motivation (BIS), which reflects heightened sensitivity to threat cues [69,70], may increase pain-focused attention, hypervigilance, and avoidance behaviour. Consistent with the fear avoidance model, avoidance may reduce activity in the short term but contribute to greater disability and poorer functioning over time [4,29]. The present findings extend the literature by suggesting that dispositional avoidance motivation may underpin these behavioural patterns and contribute to pain outcomes. The consistency of the predictive effects of avoidance motivation across pain outcomes further highlights this as an important factor within biopsychosocial models.

The absence of a significant independent predictive effect of approach motivation (BAS) on pain outcomes supports the view that approach motivation is focused on reward sensitivity, positive affect, and goal-directed behaviour [21], thus more strongly related to psychological well-being and engagement than to pain outcomes per se [71]. Individuals high in approach motivation may continue to pursue valued goals and maintain positive affect despite ongoing pain, without necessarily reporting reduced pain levels. Thus, approach motivation may have a protective or buffering effect in relation to pain intensity and interference. Indeed, enhancing forms of approach motivation has become a strategy for pain management interventions when all medical avenues of pain amelioration have been explored and optimised [72]. Notably, research examining approach motivation in chronic pain remains limited, with most work focusing on avoidance-related processes. The few studies that have investigated approach motivation to pain outcomes have been inconsistent and generally weaker than those observed for avoidance motivation [71,73]. Moreover, the influence of approach motivation may be attenuated in the presence of stronger avoidance tendencies under threat [69,70]. Together, these findings suggest that approach motivation may play a more protective role in maintaining well-being and engagement in the context of ongoing pain, rather than impacting pain intensity or pain interference.

The goal disengagement findings in relation to both pain intensity and pain interference are consistent with the goal adjustment model [74], which proposes that effectively disengaging from unattainable goals reduces persistent goal outcome discrepancies and the strain of repeated failure. In chronic pain, where valued goals may become constrained or unattainable, effective disengagement may reduce pressure to persist with goals that cannot be achieved under current pain-related limitations. Theoretically, it is thought that effectively giving up goals that can no longer be reached is protective of mental well-being [75]. In the case of chronic pain, disengaging from an unattainable goal may reduce unnecessary mental and physical effort. This may, in turn, support more effective pacing and reduce unsustainable activity that could trigger pain flare-ups. In chronic pain research, goal disengagement has been examined more often in relation to psychological adaptation and mental well-being than in relation to pain outcomes directly [48,76]. The present findings extend the literature by indicating that effective goal disengagement is also implicated in reduced pain intensity and pain interference.

The findings that greater goal re-engagement predicted increased pain interference were unexpected. Although goal re-engagement is theoretically considered adaptive, reflecting investment in alternative meaningful goals that support adjustment [74], prior research has primarily linked it to improved psychological well-being and purpose in life [48,77] rather than to reductions in pain interference or intensity. As such, the present finding is not necessarily inconsistent with the literature, as goal re-engagement may retain an adaptive role in supporting well-being while relating differently to pain interference. One possible interpretation is that, in chronic pain, greater re-engagement may not always reflect successful adaptation but continued investment in multiple or alternative goals despite ongoing pain-related limitations. For example, taking on new goals while still managing substantial pain demands may increase disruption across daily activities and, in turn, perceived pain interference. Further, the often fluctuating and inconsistent nature of pain conditions may require that individuals go through a continual process of ‘relearning’ their physical limits and capabilities. In clinical contexts, this process is often described as trial and error, as individuals attempt to learn their limits under fluctuating pain conditions, where boundaries may shift over time. Thus, re-engagement may support meaning and longer-term adjustment while still coinciding with greater short-term interference when pain continues to constrain goal pursuit. The present findings, therefore, suggest that goal re-engagement might still help psychological adjustment such as well-being, but that does not mean it will also help pain interference.

Pain-specific goal strategies focus specifically on accommodative (meaningfulness of life despite pain, acceptance of the insolubility of pain) and assimilative (solving pain, and belief in a solution) strategies. The significant ‘pain solution’ findings may indicate that in chronic pain, when pain is often not fully controllable, persistent failed attempts to “solve” pain may sustain frustration and symptom focus. For example, individuals high in ‘solving pain’ may repeatedly focus on finding ways to eliminate pain, investing time and limited energy resources in the pursuit, which, in turn, may increase pain and the extent to which pain is experienced as interfering. This interpretation is consistent with previous research, which suggests persistent efforts to solve or eliminate pain are more closely linked to distress and maladjustment than to better pain outcomes [40,78]. These findings suggest that pain management strategies may benefit from supporting individuals not only in attempts to reduce pain but also in developing ways of living well alongside pain when it is not fully controllable.

The fact the results showed that the accommodative strategy of maintaining a meaningful life despite pain significantly predicted lower interference but not intensity is consistent with the broader acceptance-based research showing that valued living is more closely linked to lower disability and better functioning than to reduced pain intensity itself [40,78]. In contrast, acceptance of the insolubility of pain independently predicted higher pain intensity and pain interference, which is not the pattern typically expected for the accommodative strategy. One plausible interpretation is that this subscale may, in some cases, reflect resignation to severe or intrusive pain rather than adaptive acceptance per se. This interpretation is supported by content-analytic work showing that chronic pain “acceptance” measures do not all assess the same construct and that disengagement from pain control, pain willingness, and engagement in activities despite pain are separable components [79]. It is also possible that individuals experiencing greater pain intensity and interference may increasingly adopt acceptance-related coping responses, over time, as a consequence of persistent pain burden, rather than acceptance contributing to poorer pain outcomes. Although our hypotheses were theoretically informed, given the cross-sectional nature of the present study, the directionality of this relationship remains unclear. Taken together, these findings suggest that, in chronic pain, the most adaptive pain-specific goal strategies for pain interference may be maintaining meaning and engagement despite pain, whereas persistent efforts to solve pain, and possibly some forms of accepting that pain cannot be solved, may be linked to greater pain burden.

Our findings have practical and public health relevance. For instance, the findings indicate that it would be beneficial for clinicians to adopt a sensitive, person-centred approach in their interactions to engender a sense of realistic hope within individuals in the context of there being no known cure or “fix”. Clinically, the findings indicate that chronic pain assessment and intervention would benefit from the inclusion of personal motivational sensitivities and adaptive goal processes and strategies. For example, psychologically informed pain management approaches may help individuals identify maladaptive avoidance patterns and heightened avoidance sensitivity, strengthen flexible goal adjustment, and support engagement in meaningful and valued activities despite ongoing pain. Clinically, it may also help to bolster approach motivation associated with meaningful reward or accomplishment. For example, rather than avoiding physical activity entirely due to anticipated pain, individuals may benefit from gradually engaging in smaller and more manageable activities (e.g., brief periods of walking), which may help strengthen adaptive approach tendencies while reducing excessive avoidance over time. Motivational and goal processes, while relatively stable constructs, are amendable to change. Therefore, the ongoing development of effective future pain interventions that take into account a motivational perspective may show promise in promoting effective adaptations, functioning and well-being for people living with chronic pain.

### Limitations and Future Research

Key methodological limitations deserve comment. First, the cross-sectional design precludes causal inference; therefore, the findings should not be interpreted as causal. Although the hypotheses were theoretically informed, it is also possible that pain influences motivational and goal regulation processes. It remains possible that greater pain influences motivational and goal regulatory processes rather than the reverse. Second, all variables were assessed using self-report questionnaires administered during a single online session, which may have increased the risk of common method variance and shared response biases, potentially inflating associations among study variables. Third, the relatively low to modest internal consistency observed for the FFFS and goal disengagement subscales represents a methodological limitation. The use of more reliable measures of these constructs may result in a different pattern of findings. Fourth, the findings for some constructs, particularly goal re-engagement and acceptance of pain’s insolubility, may be context-dependent and should, therefore, be interpreted cautiously. Fifth, the inclusion of heterogeneous chronic pain conditions, including both primary and secondary pain presentations, may have introduced variability related to differences in underlying pathology, prognosis, treatment burden, and psychological adjustment, which could be associated motivational and goal regulation processes. Future research would benefit from prospective longitudinal designs to ascertain whether motivation and goal regulation processes precede or follow pain-related changes over time. Given the relatively limited research on approach motivation in chronic pain, further investigation of approach motivation and its association with mental well-being is warranted. Further, future research could examine whether distinct goal regulation processes play a role in explaining the relationship between approach motivation and mental well-being for people living with pain. The present research examined chronic pain broadly, given the relatively novel nature of this area of research. However, future research could examine whether distinct motivational and goal regulation processes differ across specific chronic pain categories, including cancer-related pain and distinct primary versus secondary chronic pain conditions.

## 5. Conclusions

In conclusion, the present study suggests that motivational orientation, adaptive goal processes, and pain-specific goal adaptive strategies are differentially associated with pain intensity and pain interference in chronic pain. Avoidance motivation emerged as the clearest predictor of worse pain outcomes, whereas goal disengagement and maintaining meaning despite pain were linked to reduced pain burden. In contrast, goal re-engagement, persistent solving efforts, and acceptance of pain’s insolubility were associated with greater pain interference and pain severity, highlighting the complexity of motivational sensitivity and goal regulation in chronic pain.

## Figures and Tables

**Table 1:** Demographic characteristics of the sample.

Variable	N (%)
Sex	
Male	94 (49.5)
Female	96 (50.5)
Education level	
Primary/Secondary	63 (33.2)
TAFE/Diploma	46 (24.2)
Tertiary/University	81 (42.6)
Employment status	
Employed full-time	81 (42.6)
Employed part-time	25 (13.2)
Sick leave	1 (0.5)
Carer	8 (4.2)
Retired	48 (25.3)
Unemployed	14 (7.4)
Student/Part-time	5 (2.6)
Other	8 (4.2)
Marital status	
Single	54 (28.4)
Married/Partner	110 (57.9)
Separated/Divorced	16 (8.4)
Widowed	10 (5.3)
Mean Age (SD)	49.46 (17.38)

**Table 2:** Summary of study measures and response scales.

Measure	Authors (Year)	Construct	Items	Response Scale
Motivation				
RST-PQ-S	Vecchione & Corr (2021) [53]	BIS, BAS (RI, GDP, RR, Imp), FFFS	22	4-point Likert
Goal Processes				
TEN/FLEX	Brandtstädter & Renner (1990) [38]	TGP, FGA	30	5-point Likert
GAS	Wrosch et al. (2003) [37]	Goal disengagement, goal reengagement	10	5-point Likert
PaSol	De Vlieger et al. (2006) [40]	Meaningfulness of life despite pain, acceptance of the insolubility of pain, belief in a solution, solving pain	14	7-point Likert
Pain				
NRS	Downie et al. (1978) [54]	Pain intensity	1	0–10 rating
PDI	Pollard (1984) [55]	Pain-related disability/interference	7	0–10 rating
CPGS	Von Korff et al. (1992) [56]	Pain intensity, pain interference, disability days	7	0–10 rating

**Note.** RST-PQ-S = Reinforcement Sensitivity Theory of Personality Questionnaire Short Form; BIS = Behavioural Inhibition System; BAS = Behavioural Activation System; RI = Reward Interest; GDP = Goal-Drive Persistence; RR = Reward Reactivity; Imp = Impulsivity; FFFS = Fight–flight–freeze System; TEN/FLEX = Tenacious Goal Pursuit and Flexible Goal Adjustment Scale; TGP = Tenacious Goal Pursuit; FGA = Flexible Goal Adjustment; GAS = Goal Adjustment Scale; PaSol = Pain Solutions Questionnaire; NRS = Numeric Rating Scale; PDI = Pain Disability Index; CPGS = Chronic Pain Grade Scale.

**Table 3:** Pain characteristics of sample (N = 190).

Variable	N (%)
Pain type	
Musculoskeletal (e.g., back pain)	113 (33.5)
Neuropathic (nerve pain)	49 (14.5)
Inflammatory (e.g., rheumatoid arthritis)	46 (13.6)
Headache/migraine pain	56 (16.6)
Choice Facial pain	10 (3)
Visceral pain (e.g., abdominal pain)	18 (5.3)
Post-surgical or injury-related pain	14 (4.2)
Fibromyalgia/ widespread pain	11 (3.3)
Cancer-related pain	10 (3)
Other	9 (2.7)
Prefer not to say	1 (0.3)
Pain duration	
3–6 months	47 (24.7)
6–12 months	31 (16.3)
1–2 years	22 (11.6)
3–5 years	29 (15.3)
>5 years	61 (32.1)
Current pain intensity	
0–2 No to little pain	19 (10)
3–5 Low to moderate	53 (27.9)
6–8 Severe	100 (52.6)
9–10 Very severe to worse pain ever	18 (9.5)
Chronic pain grade	
Grade I (low disability, low intensity)	56 (29.5)
Grade II (low disability, high intensity)	49 (25.8)
Grade III (high disability, moderately limiting)	51 (26.8)
Grade IV (high disability, severely limiting)	34 (17.9)
Disability days, Mean (SD)	21.57 (38.9)

**Table 4:** Descriptive statistics and internal consistency of study variables.

Variable	M	SD	Range
Pain			
Pain Intensity (NRS)	5.78	2.17	1–10
Pain Disability Index (PDI)	34.66	15.21	1–70
Pain Intensity (CPGS)	57.77	20.78	7–100
Pain Interference (CPGS)	49.96	24.99	0–100
Disability Days (CPGS)	21.57	38.90	0–180
Goal Processes			
Tenacious Goal Pursuit (TGP)	46.88	7.80	25–72
Flexible Goal Adjustment (FGA)	50.13	7.35	25–72
Goal Disengagement	11.38	2.81	4–19
Goal Reengagement	19.76	4.74	6–30
PaSol Goal Strategy Processes			
Solving Pain	15.18	4.77	4–24
Meaningfulness of Life Despite Pain	18.81	5.79	0–30
Acceptance of the Insolubility of Pain	9.63	4.05	0–18
Belief in a Solution	6.72	3.10	0–12
Motivational Systems (BIS/BAS)			
BIS	13.77	3.15	5–20
BAS Reward Interest	7.45	2.27	3–12
BAS Goal Drive Persistence	8.36	2.20	3–12
BAS Reward Reactivity	8.66	2.03	3–12
BAS Impulsivity	7.28	2.36	3–12
BAS Total	31.75	6.84	12–48
FFFS	13.16	3.16	5–20

NRS = Numerical Rating Scale for; CPGS = Chronic Pain Grade Scale; BIS = Behavioural Inhibition System; BAS = Behavioural Activation System; FFFS = Fight–flight–freeze System.

**Table 5:** Correlations between pain intensity, pain interference, and motivational orientations, goal processes and strategies.

	1	2	3	4	5	6	7	8	9	10	11	12	13	14	15	16	17	18
1. NRS	1																	
2. Pain Intensity	0.69 ***	1																
3. PDI	0.51 ***	0.59 ***	1															
4. Pain Interfer	0.57 **	0.76 ***	0.68 ***	1														
5. BIS	0.23 **	0.27 ***	0.28 ***	0.36 ***	1													
6. BAS RI	0.08	0.18 *	0.13	0.17 *	0.30 ***	1												
7. BAS GDP	0.08	0.12	0.10	0.10	0.10	0.59 ***	1											
8. BAS RR	0.04	0.14	0.15 *	0.15 *	0.38 ***	0.58 ***	0.47 ***	1										
9. BAS Imp	0.10	0.20 **	0.29 ***	0.29 ***	0.46 ***	0.48 ***	0.22 **	0.44 ***	1									
10. BAS Total	0.10	0.21 **	0.22 **	0.23 **	0.40 ***	0.86 ***	0.73 ***	0.80 ***	0.71 ***	1								
11. FFFS	−0.02	0.09	0.12	0.07	0.30 ***	0.25 ***	0.23 **	0.52 ***	0.19 **	0.38 ***	1							
12. TGP	−0.06	−0.08	−0.16 *	−0.11	−0.25 ***	0.23 **	0.41 ***	0.06	−0.09	0.20 **	−0.11	1						
13. FGA	−0.15 *	−0.11	−0.13	−0.13	−0.30 ***	0.26 ***	0.28 ***	0.16 *	−0.06	0.20 **	−0.01	0.34 ***	1					
14. Goal Dis	−0.12	−0.14	−0.01	−0.09	0.03	−0.17 *	−0.33 ***	−0.09	−0.01	−0.22 **	−0.06	−0.49 ***	−0.07	1				
15. Goal Re	−0.03	0.04	0.08	0.12	0.03	0.30 ***	0.27 ***	0.30 ***	0.19 *	0.34 ***	0.14 *	0.02	0.44 ***	0.01	1			
16. Solving pain	0.12	0.16 *	0.08	0.13	0.10	0.34 ***	0.38 ***	0.42 ***	0.24 ***	0.45 ***	0.24 ***	0.15 *	0.12	−0.23 ***	0.11	1		
17. Meaningfns	−0.07	0.03	−0.06	−0.08	−0.06	0.49 ***	0.43 ***	0.41 ***	0.19 **	0.49 ***	0.20 **	0.28 ***	0.49 ***	−0.13	0.42 ***	0.30 ***	1	
18. Acceptance	0.17 *	0.17 *	0.18 *	0.18 *	0.07	0.33 ***	0.22 **	0.20 **	0.20 **	0.31 ***	0.06	0.10	0.23 **	0.02	0.31 ***	0.04	0.50 ***	1
19. Belief	−0.03	0.01	−0.01	0.01	−0.01	0.47 ***	0.35 ***	0.47 ***	0.30 ***	0.51 ***	0.22 **	0.17 *	0.34 ***	−0.14	0.33 ***	0.54 ***	0.49 ***	0.09

**Note**. Significance level = * *p* < 0.05; ** *p* < 0.01; *** *p* < 0.001; Unmarked correlations are non-significant; (2-tailed); N = 190. 1. NRS = Numerical Rating Scale for Pain Intensity; 2. Pain Intensity = Pain Intensity (Chronic Pain Grade Scale); 3. PDI = Pain Disability Index; 4. Pain Interfer = Pain Interference (Chronic Pain Grade Scale); 5. BIS = Behavioural Inhibition System; 6. BAS RI = Reward Interest; 7. BAS GDP = BAS Goal-Drive Persistence; 8. BAS RR = BAS Reward Reactivity; 9. BAS Imp = BAS Impulsivity; 10. BAS Total = Behavioural Activation System Total; 11. FFFS = Fight–flight–freeze System; 12. TGP = Tenacious Goal Pursuit Scale; 13. FGA = Flexible Goal Adjustment Scale; 14. Goal Dis = Goal Disengagement (GAS); 15. Goal Re = Goal Reengagement (GAS); 16. Solving pain (PaSol); 17. Meaningfns = Meaningfulness of Life Despite Pain (PaSol); 18. Acceptance = Acceptance of the Insolubility of Pain (PaSol); 19. Belief = Belief in a Solution (PaSol).

**Table 6:** Hierarchical regression predicting pain intensity from motivational orientations (controlling for age and gender).

	Variable	*B*	*SE*	*β*	*t*	*p*	95% CI		*R* ^2^	Δ*R* ^2^	Δ*F*(*p*)	*F*(*p*)
							LL	UL				
Step 1									0.006	0.01	0.59	0.59
	Constant	57.48	2.16		26.64	<0.001	53.22	61.73				
	Age	0.10	0.09	0.08	1.09	0.278	−0.08	0.27				
	Gender	0.58	3.05	0.01	0.19	0.849	−5.43	6.60				
Step 2									0.11	0.10	7.18 (<0.001)	4.57 (<0.001)
	Constant	22.28	8.77		2.54	0.012	4.97	39.59				
	Age	0.20	0.09	0.17	2.29	0.023	0.03	0.37				
	Gender	−1.01	3.08	−0.02	−0.33	0.742	−7.10	5.07				
	BIS	1.67	0.52	0.25	3.22	0.002	0.64	2.69				
	BAS	0.48	0.25	0.16	1.93	0.055	−0.01	0.97				
	FFFs	−0.16	0.53	−0.02	−0.30	0.765	−1.19	0.88				

**Note**. *B* = unstandardised regression coefficient; *SE* = standard error; *β* = standardised regression coefficient; CI = confidence interval; LL = lower limit; UL = upper limit; *R*^2^ = proportion of variance explained; Δ*R*^2^ = change in explained variance; Δ*F* = change in F statistic; BIS = Behavioural Inhibition System; BAS = Behavioural Activation System; FFFS = Fight–Flight–Freeze System.

**Table 7:** Hierarchical regression predicting pain interference from motivational orientations (controlling for age and gender).

	Variable	*B*	*SE*	*β*	*t*	*p*	95% CI		*R* ^2^	Δ*R* ^2^	Δ*F*(*p*)	*F*(*p*)
							LL	UL				
Step 1									0.02	0.02	1.86	1.86
	Constant	51.02	2.58		19.80	<0.001	45.94	56.11				
	Age	−0.20	0.10	−0.14	−1.90	0.059	−0.41	0.01				
	Gender	−2.09	3.64	−0.04	−0.57	0.567	−9.27	5.09				
Step 2									0.15	0.13	9.52 (<0.001)	6.56 (<0.001)
	Constant	7.32	10.3		0.71	0.478	−13.01	27.65				
	Age	−0.07	0.10	−0.05	−0.65	0.518	−0.269	0.14				
	Gender	−4.26	3.62	−0.09	−1.18	0.241	−11.41	2.88				
	BIS	2.66	0.61	0.34	4.38	<0.001	1.46	3.86				
	BAS	0.40	0.29	0.11	1.37	0.173	−0.18	0.97				
	FFFs	−0.34	0.62	−0.04	−0.55	0.585	−1.55	0.88				

**Note**. *B* = unstandardised regression coefficient; *SE* = standard error; *β* = standardised regression coefficient; CI = confidence interval; LL = lower limit; UL = upper limit; *R*^2^ = proportion of variance explained; Δ*R*^2^ = change in explained variance; Δ*F* = change in F statistic; BIS = Behavioural Inhibition System; BAS = Behavioural Activation System; FFFS = Fight–Flight–Freeze System.

**Table 8:** Hierarchical regression predicting pain intensity from goal processes and strategies (controlling for age and gender).

	Variable	*B*	*SE*	*β*	*t*	*p*	95% CI		*R* ^2^	Δ*R* ^2^	Δ*F*(*p*)	*F*(*p*)
							LL	UL				
Step 1									0.01	0.00	0.59	0.59
	Constant	57.48	2.16		26.64	<0.001	53.22	61.73				
	Age	0.10	0.09	0.08	1.09	0.278	−0.08	0.27				
	Gender	0.58	3.05	0.01	0.19	0.849	−5.43	6.59				
Step 2									0.07	0.04	3.26	2.38
	Constant	104.7	16.36		6.40	<0.001	72.43	136.99			(0.013)	(0.031)
	Age	0.14	0.09	0.11	1.54	0.126	−0.04	0.31				
	Gender	0.24	3.02	0.01	0.08	0.935	−5.71	6.20				
	TGP	−0.41	0.23	−0.16	−1.76	0.079	−0.86	0.05				
	FGA	−0.39	0.24	−0.14	−1.60	0.110	−0.87	0.09				
	Goal Dis	−1.70	0.61	−0.23	−2.81	0.005	−2.90	−0.51				
	Goal Re	0.57	0.36	0.13	1.60	0.112	−0.14	1.28				
Step 3									0.14	0.09	3.51	2.92
	Constant	90.7	17.01		5.33	<0.001	55.13	124.28			(0.009)	(0.002)
	Age	0.15	0.09	0.12	1.65	0.100	−0.02	0.32				
	Gender	2.24	3	0.05	0.75	0.456	−3.68	8.17				
	TGP	−0.43	0.23	−0.16	−1.88	0.062	−0.88	0.02				
	FGA	−0.40	0.25	−0.14	−1.59	0.114	−0.89	0.10				
	Goal Dis	−1.55	0.60	−0.21	−2.56	0.011	−2.74	−0.36				
	Goal Re	0.33	0.37	0.07	0.87	0.384	−0.41	1.06				
	Solving pain	0.80	0.37	0.18	2.16	0.032	−0.07	1.53				
	Meaningful	−0.31	0.37	−0.09	−0.85	0.395	−1.03	0.41				
	Acceptance	1.29	0.43	0.25	2.97	0.003	0.43	2.15				
	Belief	−0.21	0.66	−0.03	−0.32	0.749	−1.50	1.09				

Note. *B* = unstandardised regression coefficient; *SE* = standard error; *β* = standardised regression coefficient; CI = confidence interval; LL = lower limit; UL = upper limit; *R*^2^ = proportion of variance explained; Δ*R*^2^ = change in explained variance; Δ*F* = change in F statistic; TGP = Tenacious Goal Pursuit; FGA = Flexible Goal Adjustment; Goal Dis = Goal Disengagement; Goal Re = Goal Re-engagement; Solving pain = Solving pain (PaSol subscale); Meaningful = Meaningfulness of Life Despite Pain (PaSol subscale); Acceptance = Acceptance of the Insolubility of Pain (PaSol subscale); Belief = Belief in a Solution (PaSol subscale).

**Table 9:** Hierarchical regression predicting pain interference from goal processes and strategies (controlling for age and gender).

	Variable	*B*	*SE*	*β*	*t*	*p*	95% CI		*R* ^2^	Δ*R* ^2^	Δ*F*	*F*
							LL	UL				
Step 1									0.02	0.02	1.86	1.86
	Constant	51.02	2.58		19.80	<0.001	45.94	56.11				
	Age	−0.20	0.11	−0.14	−1.90	0.059	−0.41	0.01				
	Gender	−2.09	3.64	−0.04	−0.57	0.567	−9.27	5.09				
Step 2									0.10	0.08	3.83 (0.005)	2.98 (0.005)
	Constant	101.84	19.43		5.24	<0.001	60.51	140.18				
	Age	−0.14	0.10	−0.09	−1.30	0.196	−0.34	0.07				
	Gender	−2.89	3.60	−0.06	−0.81	0.421	−9.96	4.18				
	TGP	−0.48	0.29	−0.15	−1.73	0.086	−1.03	0.07				
	FGA	−0.59	0.29	−0.17	−2.03	0.044	−1.16	0.02				
	Goal Dis	−1.74	0.72	−0.20	−2.42	0.017	−3.16	−0.32				
	Goal Re	1.08	0.42	0.21	2.53	0.012	0.24	1.92				
Step 3									0.18	0.08	4.55 (0.002)	3.89 (<0.001)
	Constant	82.73	19.98		4.14	<0.001	43.29	122.18				
	Age	−0.11	0.10	−0.08	−1.09	0.276	−0.32	0.10				
	Gender	−0.39	3.53	−0.01	−0.11	0.912	−7.35	6.57				
	TGP	−0.43	0.27	−0.14	−1.60	0.112	−0.97	0.10				
	FGA	−0.42	0.30	−0.13	−1.44	0.152	−1.01	0.16				
	Goal Dis	−1.68	0.71	−0.19	−2.37	0.019	−3.08	−0.28				
	Goal Re	1.00	0.44	0.19	2.29	0.023	0.14	1.87				
	Solving pain	0.92	0.44	0.18	2.13	0.035	−0.07	1.78				
	Meaningful	−1.17	0.43	−0.27	−2.73	0.007	−2.02	−0.33				
	Acceptance	1.81	0.51	0.29	3.55	<0.001	0.80	2.81				
	Belief	−0.18	0.77	−0.02	−0.23	0.816	−1.70	1.34				

**Note**. *B* = unstandardised regression coefficient; *SE* = standard error; *β* = standardised regression coefficient; CI = confidence interval; LL = lower limit; UL = upper limit; *R*^2^ = proportion of variance explained; Δ*R*^2^ = change in explained variance; Δ*F* = change in F statistic; TGP = Tenacious Goal Pursuit; FGA = Flexible Goal Adjustment; Goal Dis = Goal Disengagement; Goal Re = Goal Re-engagement; Solving pain = Solving pain (PaSol subscale); Meaningful = Meaningfulness of Life Despite Pain (PaSol subscale); Acceptance = Acceptance of the Insolubility of Pain (PaSol subscale); Belief = Belief in a Solution (PaSol subscale).

## Data Availability

The data are not publicly available as the dataset forms part of an ongoing research programme. An anonymised electronic dataset will be available from the corresponding author upon reasonable request.

## Supplementary Materials

The following supporting information can be downloaded at: https://www.mdpi.com/article/10.3390/ijerph23060708/s1, Text S1: Regression analysis for alternative pain intensity and interference NRS and PDI; Text S2: Motivation orientations in relation to pain intensity and pain interference; Text S3: Adaptive goal processes and pain-specific goal strategies in relation to pain intensity and interference; Text S4: Sensitivity analyses for cancer pain and non-cancer pain; Table S1: Hierarchical regression predicting NRS pain intensity from motivational orientations (controlling for age and gender); Table S2: Hierarchical regression predicting PDI pain interference from motivational orientations (controlling for age and gender); Table S3: Hierarchical regression predicting NRS pain intensity from goal processes and strategies (controlling for age and gender); Table S4: Hierarchical regression predicting PDI pain interference from goal processes and Strategies (controlling for age and gender). Ref. [1] is cited in Supplementary Material.
