# Supplementary material for: Approach–Avoidance Motivation and Goal Adaptation in Chronic Pain: Predicting Pain Intensity and Interference
Source: Int J Environ Res Public Health. 2026 May 27;23(6):708. doi: 10.3390/ijerph23060708 (PMC13299446; doi:10.3390/ijerph23060708)
Supplement: Supplementary file 1 [file ijerph-23-00708-s001.zip › ijerph-4304273-supplementary.pdf]

Supplementary Analyses

Regression analysis for alternative pain intensity and interference NRS, and PDI

To complement the main study findings, further supplementary analyses were conducted to investigate whether the main motivation, adaptive goal processes and pain-specific goal strategies showed the same significant and non-significant effects when using two alternative measures of pain intensity (NRS) and pain interference (PDI), respectively. For the most part, the significant and non-significant effects remained the same.

Motivation orientations in relation to pain intensity and pain interference

As shown in Tables S1 and S2, at the motivational level, avoidance motivation (BIS), but not approach motivation (BAS), remained a significant independent predictor of both pain intensity and pain interference. This pattern of results is consistent with the main study findings. That is, increased avoidance motivation (but not approach motivation) positively predicted both pain intensity and pain interference.

Table S1. Hierarchical regression predicting NRS pain intensity from motivational orientations (controlling for age and gender).

	Variable	*B*	*SE*	*β*	*t*	*p*	95% CI		R^2	ΔR^2	$\Delta F(p)$	$F(p)$
							LL	UL				
Step 1									0.04	0.04	3.66	3.66
	Constant	6.20	0.22		27.98	<0.001	5.76	6.64			(0.028)	(0.028)
	Age	0.00	0.01	0.01	0.18	0.860	−0.02	0.019				
	Gender	−0.83	0.31	−0.19	−2.65	0.009	−1.45	−0.21				
Step 2									0.12	0.08	5.460	4.84
	Constant	3.61	0.91		3.96	<0.001	1.81	5.41			(0.001)	(0.001)
	Age	0.01	0.01	0.08	1.06	0.292	−0.01	0.03				
	Gender	−0.97	0.32	−0.23	−3.04	0.003	−1.61	−0.34				
	BIS	0.20	0.05	0.29	3.69	<0.001	0.09	0.31				
	BAS	0.01	0.03	0.03	0.37	0.710	−0.04	0.06				
	FFFs	−0.03	0.06	−0.04	−0.53	0.599	−0.14	0.08				

Note. *B* = unstandardised regression coefficient; *SE* = standard error; *β* = standardised regression coefficient; CI = confidence interval; LL = lower limit; UL = upper limit; R^2 = proportion of variance explained; ΔR^2 = change in explained variance; ΔF = change in F statistic; BIS = Behavioural Inhibition System; BAS = Behavioural Activation System; FFFS = Fight–Flight–Freeze System.

Table S2. Hierarchical regression predicting PDI pain interference from motivational orientations (controlling for age and gender).

	Variable	*B*	*SE*	*β*	*t*	*p*	95% CI		R^2	ΔR^2	ΔF	*F*
							LL	UL				
Step 1									0.01	0.01	0.90	0 .90
	Constant	34.13	1.58		21.65	<.001	31.02	37.24				
	Age	−0.08	0.06	−0.09	−1.19	0.237	−0.20	0.05				
	Gender	1.04	2.23	0.03	0.47	0.640	−3.35	5.44				
Step 2									0.09	0.08	5.60**	3.74**
	Constant	10.42	6.49		1.61	0.110	−2.37	23.22				
	Age	−0.01	0.07	−0.01	−0.17	0.863	−0.14	0.12				

Gender	−0.31	2.28	−0.01	−0.14	0.891	−4.81	4.18
BIS	1.11	0.38	0.23	2.89	0.004	0.35	1.86
BAS	0.26	0.18	0.12	1.44	0.153	−0.10	0.62
FFFs	0.06	0.39	0.01	0.16	0.873	−0.70	0.83

$p < .01$. **Note. *B* = unstandardised regression coefficient; *SE* = standard error; *β* = standardised regression coefficient; CI = confidence interval; LL = lower limit; UL = upper limit; R^2 = proportion of variance explained; ΔR^2 = change in explained variance; ΔF = change in F statistic; BIS = Behavioural Inhibition System; BAS = Behavioural Activation System; FFFs = Fight–Flight–Freeze System.

Adaptive goal processes & pain-specific goal strategies in relation to pain intensity and interference

At the goal level of analyses, and as shown in Tables S3 and S4, the findings at the goal level of analysis when using the two different measures of pain (NRS, PDI) than used in the main study. Again, at the goal level of analysis the significant effect, and for the most part, the non-significant effects remained that same as those reported in the main study. That is, greater goal disengagement independently and significantly predicted reduced pain intensity. Meaningfulness independently and significantly predicted reduced pain intensity and pain interference. Acceptance of the insolubility of pain again independently and significantly predicted increased pain intensity and pain interference. The non-significant findings for most part remained the same as those reported in the main study results, except that goal disengagement and goal re-engagement did not reach significance in relation to pain interference. Gender was found to be a significant predictor of pain intensity, (but not in the main study). The gender results indicate that men, relative to women, were more inclined to report increased pain intensity.

Table S3. Hierarchical regression predicting NRS pain intensity from goal processes and strategies (controlling for age and gender).

	Variable	*B*	*SE*	*β*	*t*	*p*	95% CI		R^2	ΔR^2	$\Delta F(p)$	$F(p)$
							LL	UL				
Step 1									0.04	0.04	3.66 (0.028)	3.66 (0.028)
	Constant	6.20	0.22		27.98	<0.001	5.76	6.64				
	Age	0.00	0.01	0.01	0.177	0.860	−0.02	−0.02				
	Gender	−0.83	0.31	−0.19	−2.65	0.009	−1.45	−0.21				
Step 2									0.09	0.05	2.52 (0.043)	3.28 (0.009)
	Constant	10.84	1.69		6.40	<0.001	7.50	14.18				
	Age	0.01	0.01	0.04	0.56	0.579	−0.01	0.02				
	Gender	−0.83	0.31	−0.19	−2.67	0.008	−1.45	−0.22				
	TGP	−0.03	0.02	−0.12	−1.38	0.170	−0.09	0.01				
	FGA	−0.05	0.03	−0.15	−1.77	0.078	−0.09	0.01				
	Goal Dis	−0.14	0.06	−0.18	−2.25	0.025	−0.27	−0.02				
	Goal Re	0.04	0.04	0.09	1.06	0.290	−0.03	0.11				
Step 3									0.15	0.07	3.51	3.42
	Constant	9.43	1.76		5.35	<0.001	5.95	12.90			(0.009)	(<0.001)
	Age	0.01	0.01	0.06	0.80	0.427	−0.01	0.03				

	Gender	−0.63	0.31	−0.15	−2.03	0.044	−1.24	−0.02
	TGP	−0.03	0.02	−0.14	−1.33	0.185	−0.08	0.02
	FGA	−0.04	0.03	−0.13	−1.46	0.146	−0.09	0.01
	Goal Dis	−0.13	0.06	−0.17	−2.13	0.035	−0.26	−0.01
	Goal Re	0.02	0.04	0.05	0.63	0.531	−0.05	0.10
	Solving pain	0.07	0.04	0.16	1.85	0.065	−0.01	0 .15
	Meaningful	−0.08	0.04	−0.20	−1.99	0.048	−0.15	−0.00
	Acceptance	0.15	0.05	0.27	3.25	0.001	0.00	0.23
	Belief	0.00	0.07	0.01	0.05	0.958	−0.14	0.14

Note. *B* = unstandardised regression coefficient; *SE* = standard error; *β* = standardised regression coefficient; CI = confidence interval; LL = lower limit; UL = upper limit; R^2 = proportion of variance explained; ΔR^2 = change in explained variance; ΔF = change in F statistic; TGP = Tenacious Goal Pursuit; FGA = Flexible Goal Adjustment; Goal Dis = Goal Disengagement; Goal Re = Goal Re-engagement; Solving pain = Solving pain (PaSol subscale); Meaningful = Meaningfulness of Life Despite Pain (PaSol subscale); Acceptance = Acceptance of the Insolubility of Pain (PaSol subscale); Belief = Belief in a Solution (PaSol subscale).

Table S4. Hierarchical regression predicting PDI pain interference from goal processes and strategies (controlling for age and gender).

	Variable	*B*	*SE*	*β*	*t*	*p*	95% CI		R^2	ΔR^2	$\Delta F(p)$	$F(p)$
							LL	UL				
Step 1									0.01	0.01	0.90	0.90
	Constant	34.13	1.58		21.65	<0.001	31.02	37.24				
	Age	−0.08	0.06	−0.09	−1.19	0.237	−0.20	0.05				
	Gender	1.04	2.23	0.03	0.47	0.640	−3.35	5.44				
Step 2									0.07	0.06	2.74	2.14
	Constant	62.59	12.02		5.21	<0.001	38.87	86.30			(0.030)	(0.051)
	Age	−0.04	0.06	−0.05	−0.63	0.528	−0.17	0.09				
	Gender	0.54	2.22	0.02	0.24	0.807	−3.83	4.92				
	TGP	−0.32	0.17	−0.16	−1.85	0.065	−0.66	0.02				
	FGA	−0.31	0.18	−0.15	−1.74	0.084	−0.66	0.04				
	Goal Dis	−0.65	0.45	−0.12	−1.45	0.149	−1.52	0.23				
	Goal Re	0.49	0.26	0.15	1.86	0.064	−0.2	1.01				
Step 3												
	Constant	54.45	12.48		4.36	<0.001	29.81	79.09	0.14	0.07	3.63	2.81
	Age	−0.02	0.07	−0.03	−0.37	0.708	−0.15	0.10			(0.007)	(0.003)
	Gender	2.11	2.21	0.07	0.96	0.340	−2.24	6.46				
	TGP	−0.31	0.17	−0.16	−1.82	0.070	−0.64	0.03				
	FGA	−0.27	0.18	−0.13	−1.45	0.149	−0.63	0.10				
	Goal Dis	−0.63	0.44	−0.12	−1.43	0.155	−1.51	0.24				
	Goal Re	0.37	0.27	0.12	1.35	0.179	−0.17	0.91				
	Solving pain	0.38	0.27	0.12	1.38	0.168	−0.16	0.89				

Meaningful	−0.54	0.27	−0.21	−2.03	0.044	−1.07	−0.01
Acceptance	1.13	0.32	0.30	3.55	<0.001	0.50	1.76
Belief	0.07	0.48	0.01	0.15	0.885	−0.88	1.02

Note. *B* = unstandardised regression coefficient; *SE* = standard error; *β* = standardised regression coefficient; CI = confidence interval; LL = lower limit; UL = upper limit; R^2 = proportion of variance explained; ΔR^2 = change in explained variance; ΔF = change in F statistic; TGP = Tenacious Goal Pursuit; FGA = Flexible Goal Adjustment; Goal Dis = Goal Disengagement; Goal Re = Goal Re-engagement; Solving pain = Solving pain (PaSol subscale); Meaningful = Meaningfulness of Life Despite Pain (PaSol subscale); Acceptance = Acceptance of the Insolubility of Pain (PaSol subscale); Belief = Belief in a Solution (PaSol subscale).

Sensitivity analyses for cancer-pain and non-cancer pain

The total sample (N =190) comprised17 participants who reported cancer related pain. Because cancer-related pain represents a form of chronic secondary pain linked to an underlying disease process [1], additional supplementary sensitivity analyses were conducted to investigate whether there were any significant differences between those who reported cancer pain (n = 17) versus non-cancer pain (n = 173). A sensitivity analysis. Welch's t-tests were used due to unequal group sizes and variance assumptions; therefore, decimal degrees of freedom are reported. Group comparisons found no significant differences between the cancer and non-cancer groups on motivational orientations and goal regulation processes. However, the cancer-related group, relative to the non-cancer group, reported significantly higher current pain intensity (NRS), $t(19.35) = 2.98$, $p = .008$, pain disability (PDI), $t(20.59) = 2.82$, $p = .010$, CPGS pain intensity, $t(20.14) = 3.02$, $p = .007$, and CPGS pain interference, $t(20.12) = 2.83$, $p = .010$.

Regression analyses were re-run after excluding participants who reported cancer-related pain. Overall, the significant and non-significant motivational effects remained the same as those reported in the main study. That is, BIS remained the only significant independent predictor of both pain intensity ($\beta = .269$, $p = .001$) and pain interference ($\beta = .336$, $p < .001$), whereas BAS and FFFS remained non-significant.

Regression results also showed the same significant and non-significant effects for distinct adaptive goal processes and pain-specific goal strategies in relations to pain intensity. That is, goal disengagement ($\beta = -.203$, $p = .021$), solving pain ($\beta = .181$, $p = .042$), and acceptance of the insolubility of pain ($\beta = .276$, $p = .002$) remained significant predictors, whereas the other predictors remained non-significant. For pain interference, the same overall pattern of significant and non-significant effects also remained the same. That is, goal disengagement ($\beta = -.176$, $p = .042$), goal re-engagement ($\beta = .207$, $p = .018$), meaningfulness of life despite pain ($\beta = -.314$, $p = .003$), and acceptance of the insolubility of pain ($\beta = .314$, $p < .001$) each significantly predicted pain interference, but solving pain did not, although it was approaching significance ($p = .061$).

1. Treede, R.-D.; Rief, W.; Barke, A.; Aziz, Q.; Bennett, M. I.; Benoliel, R.; Cohen, M.; Evers, S.; Finnerup, N. B.; First, M. B.; Giamberardino, M. A.; Kaasa, S.; Korwisi, B.; Kosek, E.; Lavand'homme, P.; Nicholas, M.; Perrot, S.; Scholz, J.; Schug, S.; Smith, B. H.; Svensson, P.; Vlaeyen, J. W. S.; Wang, S.-J., Chronic pain as a symptom or a disease: the IASP Classification of Chronic Pain for the International Classification of Diseases (ICD-11). *PAIN* **2019,** 160, (1), 19-27. https://doi.org/10.1097/j.pain.0000000000001384
